# High-Intensity Intermittent Exercise Increases Fat Oxidation Rate and Reduces Postprandial Triglyceride Concentrations

**DOI:** 10.3390/nu10040492

**Published:** 2018-04-16

**Authors:** Tsung-Jen Yang, Ching-Lin Wu, Chih-Hui Chiu

**Affiliations:** 1 Department of Physical Education, National Taiwan Normal University, Taipei 106, Taiwan; andy32437@yahoo.com.tw; 2 Graduate Institute of Sports and Health Management, National Chung Hsing University, Taichung 402, Taiwan; psclw@dragon.nchu.edu.tw; 3 Graduate Program in Department of Exercise Health Science, National Taiwan University of Sport, Taichung 404, Taiwan

**Keywords:** cardiovascular disease, energy expenditure, respiratory exchange ratio, creatine kinase

## Abstract

(1) Background: This study investigated the effect of acute barehanded whole body high-intensity intermittent exercise (HIIE) and moderate intensity and continuous exercise (MICE) at the same quantity of energy expenditure on postprandial triglyceride (TG) concentrations. (2) Methods: Nine healthy males completed three trials (HIIE, MICE and control (CON)) in a random order separated by at least 14 days. After each intervention, the participants rested for 12 h and consumed a high-fat test meal on the next day. The blood samples and respiratory exchange ratio were observed in the fasted state and for 4 h after consuming the test meal. (3) Results: The HIIE had a significantly higher area under the curve of postprandial fat oxidation rate than MICE (*p* = 0.027) and CON (*p* = 0.035) and exhibited significantly lower postprandial TG concentration than the MICE and CON at 2 and 4 h after the test meal. Moreover, the HIIE displayed a higher postprandial TG concentration area under the curve than MICE (*p* = 0.013) and CON (*p* = 0.048). (4) Conclusions: The present study concluded that acute barehanded whole body HIIE could significantly lower postprandial TG concentrations. It possibly can induce a rise in the postprandial fat oxidation rate.

## 1. Introduction

Higher postprandial triglyceride (TG) concentrations significantly increase the risks of cardiovascular disease (CVD) and insulin resistance [1,2,3]. Due to the recent prevalence of high-fat meals and the fact that postprandial plasma lipid responses remain high for a few hours [4], people are in a state of high plasma lipid concentration for the majority of a day. Therefore, decreasing postprandial lipid concentrations effectively has recently received considerable attention.

A single bout of moderate intensity and continuous exercise (MICE) can reduce postprandial TG concentrations effectively the next day [5,6]. Although the reduction in postprandial plasma lipid concentrations have been attributed to the increased lipoprotein lipase activity (LPLA) in the muscles [7,8], increased insulin sensitivity, heightened post-exercise fat oxidation rate and lowered release of very-low-density lipoprotein in the liver [9], the exact cause remains unclear. Moreover, different types of exercise have yielded similar results. For example, high-intensity intermittent exercise (HIIE) reduced postprandial TG concentrations effectively the next day [10,11,12].

The energy expenditure of exercise has been identified as a key factor for the reduction in postprandial TG concentration caused by MICE [6,7]; however, a recent study on HIIE intervention revealed that HIIE (181.0–404.8 kcal), which requires less energy expenditure than MICE, could also effectively reduce postprandial TG concentrations [10,11,12]. This result contradicts those of other studies, which have suggested that the energy expenditure of exercise must reach the substantially higher level of 476.2–547.6 kcal for the said purpose [6]. Therefore, the energy expenditure of exercise may not be the primary factor for reducing postprandial plasma lipid levels. Alternatively, the different metabolic rate after exercise may explain the effect of HIIE on lowering postprandial plasma lipid concentrations [13,14]. Thus, further investigation is required to support this statement.

Previous HIIE studies were mostly been conducted using stationary bicycles (i.e., Wingate tests) [15], which involve only lower limb muscles. Therefore, whether other body muscles will achieve the same results cannot be directly inferred. An alternative exercise, Tabata training (a high intensity intermittent workout that combines multiple types of exercise) involves the simultaneous use of upper-limb, lower-limb and torso muscles [16]. There are no studies investigating the effect of a single bout of barehanded whole body HIIE on plasma lipid concentrations after a high fat meal, so the present study used a single bout of Tabata training to fill this gap.

## 2. Materials and Methods 

### 2.1. Participants

Nine healthy male participants were recruited (age 22 ± 1.3 year, height 170.1 ± 4.7 cm, weight 75.4 ± 17.5 kg, maximal oxygen consumption (VO_2max_) 42.2 ± 7.4 mL/kg/min). None of them exercised on a regular basis, had not received any exercise training or medication in the past 6 months and had not experienced hypertension, hyperlipidemia, heart-related diseases, joint-related diseases, osteoporosis or conditions that render them unsuited for exercise. After being informed thoroughly about the procedures and potential risks of the experiment, they signed a letter of consent to officially participate in the study, which was approved by the Institutional Review Board of the Cheng Ching Hospital in Taiwan.

### 2.2. Design

The participants were requested to complete three randomly-assigned two-day trials, which consist of a high intensity intermittent exercise (HIIE) trial, moderate intensity and continuous exercise (MICE) and rest (control (CON)). The HIIE trial was performed before the MICE trial because the two exercise trials were performed at the same quantity of energy expenditure. Therefore, the order of this study was randomly assigned in sequence and separated with an interval of 14 days, as HIIE→MICE→CON, HIIE→CON→MICE or CON→HIIE→MICE. In the HIIE trial, the heart rates (HRs) and energy expenditure of the participants were recorded using wrist-worn hear rate monitors (Polar Electro, Kempele, Finland). In the MICE trial, the participants exercised with an intensity of 50% VO_2max_ until reaching the same quantity of energy expenditure required by the HIIE trial.

### 2.3. Preliminary Measurements

Before the experiment, all participants were required to receive tests of running economy (RE) and submaximal oxygen uptake for assessment of their VO_2max_. Both tests were scheduled on the same date, held at least 7 days before the experiment and carried out in an afternoon; test details are described as follows.

#### 2.3.1. Running Economy

This test was conducted on a treadmill (Lode BV, Groningen, The Netherlands) operated at a distinct, but consistent speed in each stage. A session consisted of 4 stages, lasted for 12 min and started with speeds of 5–6 km/h; the gradient was initially set at 0° and increased by 2.5° every 3 min. At each stage, the oxygen uptake, oxygen partial pressure, carbon dioxide partial pressure, energy expenditure and HR per minute of the participants were recorded for analysis.

#### 2.3.2. Submaximal Oxygen Uptake

For each participant, the test for assessing VO_2max_ was performed according to the participant’s optimal RE speed (acquired from the previous test) with gradually increasing difficulty. The gradient was initially set at 0° and increased by 2.5° every 3 min until the participant was exhausted. The participant’s VO_2max_ and the HRs at each stage were recorded for analysis.

The data of the submaximal oxygen uptake test were obtained using regression and combined with the RE test data to calculate the 50% VO_2max_ speed, an intervention velocity for MICE intervention. Regression was used to estimate the corresponding values of exercise intensity, energy expenditure and HR and to calculate the energy expenditure of the exercise by using the HR data measured in the HIIE trial.

### 2.4. Protocol

Each trial lasted for 2 days. Three days before each trial, the participants were asked to avoid foods they did not normally consume to avoid an altered metabolism during the trial. They were further required to record all food and beverage consumptions and consume the same foods and beverages during these 3 days. They were also advised against excessive physical exertion and were only to engage in static activities, such as watching TV, reading and listening to music. In the afternoon of the first day of each trial, the participants arrived at the laboratory at 17:00, rested for 30 min and then engaged in an exercise (HIIE or MICE) or rest (CON) intervention. Afterward, they were provided with a standardized meal at 18:00–18:30 and were required to consume it in 20 min, after which they returned home. Participants returned to the laboratory to receive a test for their tolerance of high-fat meals after fasting for 12 h.

The participants returned to the laboratory at 07:00 the following morning, rested for 10 min and had an indwelling needle imbedded in their forearm to collect a 10-mL fasting blood sample. Next, they were provided a high-fat test meal, which was consumed in 20 min, after which the blood specimens were collected for 4 h. Moreover, 10 min prior to fasting (0 h) and to postprandial plasma sample collections at 0.5, 1, 2, 3 and 4 h, gaseous samples were collected from the resting participants to calculate their postprandial fat oxidation rate.

#### 2.4.1. The HIIE Intervention

The HIIE trial was a Tabata training program, totaling 20 min, and consisted of 4 sessions, each of which was separated by 1 min of rest [17]. Each session consisted of 2 rounds, both of which comprised a set of 4 moves. Each move included 20 s of high-intensity training and 10 s of rest. The moves for the first session were high knee run, plank punch, jumping jacks and side skaters; those for the second session were jump rope, in/out boat, line jumps and punch-ups; those for the third session were burpees, Russian twists, squats and lunges; and those for the fourth session were mountain climbers, push-ups, split squats and box jumps. The participants’ HRs were recorded during all of the Tabata exercise to calculate their energy expenditure. The average HR/VO_2_ regressions was used to calculate VO_2_ and energy expenditure predicted by the submaximal oxygen uptake test. 

#### 2.4.2. The MICE Intervention

The MICE trial was conducted at least 14 days after the HIIE trial. Before the MICE trial could commence, regression formulas derived from the previous tests were used to calculate the energy expenditure per minute at 50% VO_2max_. In this trial, the participants arrived at the laboratory in the afternoon of the first day and engaged in walking at 50% VO_2max_ until the energy expenditure reached the same quantity of the HIIE trial.

### 2.5. Oral Fat Tolerance Test

All of the meals for the oral fat tolerance test were prepared by the same researcher. The menu consisted of toast, butter, cheese, muesli and fresh cream, which provided 1.2 g/kg of fat, 1.1 g/kg of carbohydrate (CHO), 0.33 g/kg of protein (PRO) and 16.5 kcal/kg [18]. All of the data were obtained from the nutrition labels on the food packages.

### 2.6. Blood Sample Collection and Analysis

Blood samples were collected using an indwelling venous needle (Venflon 20G, Ohmeda, Sweden) and a three-way stopcock (Connecta Ltd., Helsingborg, Sweden). Each time, 10 mL of blood were collected, and the collection was timed before the meal (at 0 h) and at 0.5, 1, 2, 3 and 4 h after the meal. At the conclusion of each collection, the catheter was cleaned with 10 mL of isotonic saline to prevent blood from clotting in the catheter. The collected samples were immediately transferred into blood collection tubes containing ethylenediaminetetraacetic acid (EDTA) for centrifugation (Eppendorf 5810, Hamburg, Germany) at 4 °C and 500× *g* for 20 min. After centrifugation, the supernatant (plasma) was immediately collected and stored in a refrigerator at −80 °C to await biochemical analysis.

The plasma concentrations of TG, glycerol, non-esterified fatty acids (NEFA) and creatine kinase (CK) were determined using an automatic biochemistry analyzer (Hitachi 7020, Tokyo, Japan) and commercially-available reagents (GOD-PAP method, Randox, Ireland).

### 2.7. Statistical Analyses

The data in the present study are all presented as the means ± the standard deviation. The biochemical readings of blood samples were compared with correlated samples using two-way repeated measures analysis of variance to identify the between-group and time variations. When statistical significance was reached, Bonferroni correction was applied for post hoc testing. The significance level was established at α = 0.05. A sufficient sample size of 8 participants was calculated using G*power 3 with an alpha value of 5% and a power of 0.8 [19].

## 3. Results

### 3.1. Exercise Indices

The HR regression curve based on the 16-min submaximal oxygen uptake test revealed that the energy expenditures in the HIIE and MICE were 384.4 ± 69.9 kcal and 376.7 ± 76.9 kcal; moreover, the maximal and average HRs for the HIIE were 171.0 ± 9.1 and 140.1 ± 3.7 beats. Because the American College of Sports Medicine guidelines define intensive exercise according to a 60–89% heart rate reserve [20], the Tabata exercise we used in this study can be defined as intensive exercise. The total exercise durations were 20 min for HIIE and 36.6 ± 4.8 min for MICE (Table 1). 

### 3.2. The Fasted State on the Morning of Day 2 of Each Main Trial

Table 2 shows the fasted state on the morning of Day 2 of each main trial. There were no differences in TG, NEFA, glycerol and fat oxidation. The plasma CK concentration was higher in the HIIE trail than the MICE and CON trails.

### 3.3. Postprandial Fat Oxidation

**Figure 1:**
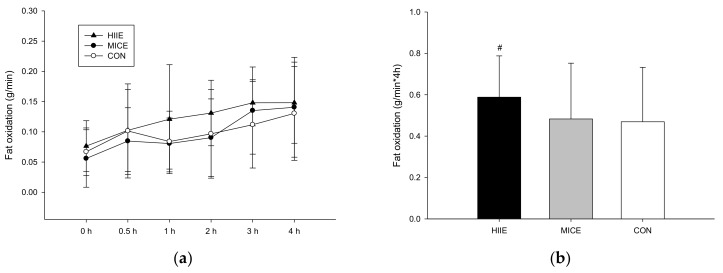
The postprandial fat oxidation rates over the 4 h (**a**) and the fat oxidation AUC (area under the curve) (**b**). # The HIIE trial was significantly higher than the MICE trial (*p* = 0.027) and the CON trial (*p* = 0.035).

The postprandial fat oxidation rates in the HIIE (Figure 1a) exhibited significant differences between trials, but no significant differences were identified in the post hoc test (trial × time, *p* = 0.544; trial, *p* = 0.034; time, *p* < 0.001). Figure 1b shows that the fat oxidation rate area under the curve (AUC) in the HIIE trial was significantly higher than the MICE trial (*p* = 0.027) and the CON trial (*p* = 0.035), but there was no significant differences between the MICE trial and the CON trial (*p* = 0.078).

### 3.4. Blood Biochemical Indices

#### 3.3.1. TG

**Figure 2:**
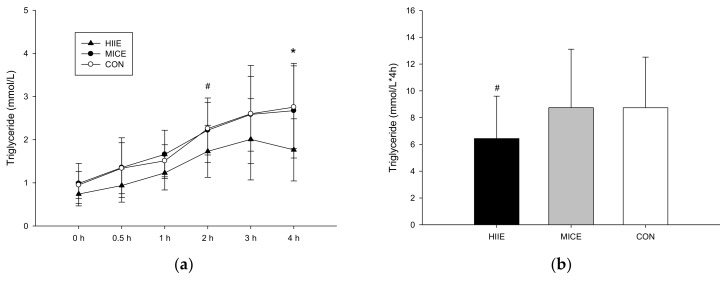
Plasma triglyceride (TG) concentrations over the 4 h during the postprandial period (**a**) and TG AUC (**b**). # Mean HIIE was significantly lower than MICE and CON after the high fat meal (*p* < 0.05). * Mean HIIE significantly was higher than CON (*p* = 0.028).

Plasma TG concentrations have significant differences between trials (trial × time, *p* = 0.196; trial, *p* = 0.012; time, *p* < 0.001; Figure 2a). At 2 and 4 h after the meal, concentrations in the HIIE trial were significantly lower than the MICE trial (2 h, *p* = 0.018; 4 h, *p* = 0.021) and the CON trial (2 h, *p* = 0.077; 4 h, *p* = 0.04). Figure 2b shows that the plasma TG AUC was lower in the HIIE trial than the MICE trial (*p* = 0.013) and the CON trial (*p* = 0.048). No significant differences in the said concentrations were found between the MICE and CON (*p* = 0.994).

#### 3.3.2. NEFA and Glycerol

**Figure 3:**
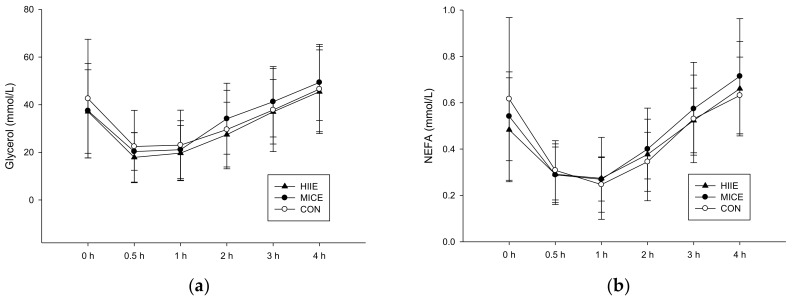
Plasma glycerol concentrations (**a**) and plasma NEFA concentrations (**b**) during the 4-h postprandial period. There were no significant differences between trials after a high fat meal.

Figure 3 shows the plasma concentrations of glycerol (a) and NEFA (b). There were no significant differences among the three trials in plasma glycerol (trial × time, *p* = 0.745; trial, *p* = 0.293; time, *p* < 0.001). Although their NEFA concentrations appeared to drop after the meal and then return to the baseline, no significant difference was observed between the three trials (trial × time, *p* = 0.574; trial, *p* = 0.701; time, *p* < 0.001).

#### 3.3.3. CK 

**Figure 4:**
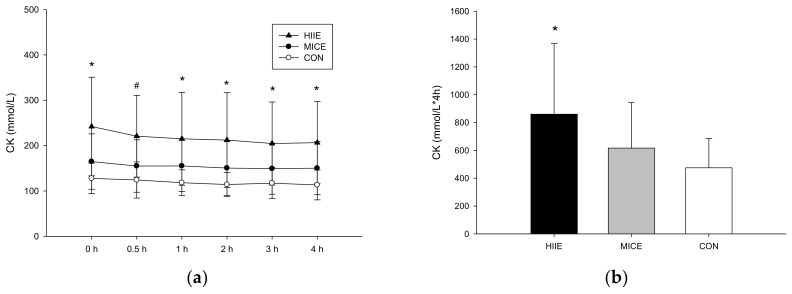
The plasma concentrations of CK (**a**) and CK AUC (**b**). # Mean HIIE significantly higher than the MICE trial and the CON trial (*p* < 0.05). * Mean HIIE significantly higher than CON (*p* < 0.05).

There were significant differences among the three trials in plasma CK (trial × time, *p* = 0.136; trial, *p* = 0.003; time, *p* < 0.001; Figure 4a). The plasma CK AUC was higher in the HIIE trial than the CON trial (*p* = 0.028; Figure 4b). There were no significant differences between the MICE trial and the CON trial (*p* = 0.098).

## 4. Discussion

The present study was the first to compare the postprandial plasma TG concentrations of barehanded whole body HIIE and MICE at the same level of energy expenditure. The results indicated that acute barehanded whole body HIIE could induce a rise in postprandial fat oxidation rate and decrease postprandial TG concentration. The similar result was consistent with previous study that found leg work type HIIE was more effective than aerobic exercise for the reduction of postprandial lipemia [21]. Moreover, the ability of exercise to lower postprandial TG concentrations was not attenuated by the inflammation resulting from muscle damage incurred through HIIE.

The postprandial fat oxidation rate was inferred to be the main reason for the significant reduction in postprandial TG concentrations in HIIE compared with MICE and CON. This result also confirmed the findings of previous studies [11,12]. Trombold et al. recruited six healthy male participants to engage in 50% VO2peak MICE for 60 min, a 90% and 25% VO2peak combination HIIE at the same energy expenditure level and rest (control trial). The HIIE reduced the postprandial TG concentrations more substantially than did the long MICE, and the postprandial fat oxidation rate of the HIIE was also significantly higher than that of the MICE [21]. However, the mechanism behind the high postprandial fat oxidation rate in HIIE remains unclear. The higher expenditure of glycogen in exercise has been considered as a possible reason for the reduction of postprandial plasma lipid level [22]; a higher intensity of exercise requires a higher expenditure of carbohydrate, particularly muscle glycogen, for energy [23,24]. Kiens and colleagues recruited participants to engage in exercise that led to glycogen exhaustion to observe the energy metabolism in the recovery period after exercise under the premise of a fixed intramyocellular triacylglycerol (IMTG) level [25]. The results indicated that the glycogen level in muscles rose significantly during an 18-h recovery period after exercise, whereas the IMTG level significantly decreased. This experiment also revealed that the respiratory exchange ratio significantly decreased during the recovery period, indicating that the carbohydrates ingested during the recovery period after exercise tend to enter the muscles, whereas the fat tends to be oxidized. Although the present study did not employ exercises that led to glycogen exhaustion, HIIE has been found to significantly induce the secretion of adrenaline during exercise. This may also explain the observation in the current study that HIIE caused a drop in the muscle glycogen level without affecting the IMTG concentrations. Moreover, similar to Kiens [26], the present study also indicated that exercise provides the benefits of raising the fat oxidation rate. Therefore, increased fat oxidation may raise the hydrolysis rate of TG after a meal, thus lowering the postprandial TG concentrations.

Another reason for the lower postprandial TG concentrations after HIIE may be the difference in excess post-exercise oxygen consumption (EPOC). Studies have indicated that at the same level of energy expenditure, the EPOC after HIIE is higher than that of low- to moderate-intensity exercise [27,28]. Moreover, the increased EPOC after anaerobic exercise continues for 14.5–24 h after exercise [27,29,30]. Although the HIIE and MICE were controlled at the same level of energy expenditure in the current study, the HIIE could still cause higher energy expenditure during the post-exercise recovery period because of its higher EPOC, thus resulting in a total energy expenditure higher than that of the MICE. Additionally, HIIE is more capable of reducing the postprandial plasma lipid concentrations than is low- to moderate-intensity exercise [31]. Thus, the difference in EPOC may also enable HIIE to substantially reduce the postprandial TG concentrations. Unfortunately, we did not measure the EPOC and 24-h energy expenditure; this is our main study limitation.

Moreover, compared with MICE, HIIE is more capable of significantly enhancing LPLA, thus causing the postprandial TG concentrations to decrease. The number of muscles that are involved in an exercise may also explain the different EPOC concentrations [32,33]. Exercise increases muscle LPLA and promotes the hydrolysis of TG into NEFA and glycerol, which are required by tissues [34,35]; the hydrolysis of TG also negatively correlates LPLA with the postprandial TG concentrations [36]. Hamilton et al. found that when exercise intensity exceeds 80% VO2max, the LPL mRNA activity in the muscles after exercise is significantly higher than lower intensity [37]. Another study required participants to use one leg for resistance exercise and found that the muscle LPLA in the exercised leg at 4–8 h after exercise was significantly higher than no exercise leg [26]. Therefore, Petitt and colleagues suggested that whole-body weight training, which can result in a higher muscle LPLA compared with MICE, is more effective at reducing the postprandial TG concentrations than partial limb training [38]. The HIIE used in the present study not only involved leg muscles (quadriceps and biceps femoris), but also pectoralis major, triceps brachii and rectus abdominis (push-ups and burpees) [17]. Because the HIIE moves involved more muscles than the MICE, HIIE may be more effective at raising muscle LPLA and, hence, more effective at lowering the postprandial TG concentrations.

The present study revealed that the postprandial CK level of the HIIE was significantly higher than MICE and CON. Studies have indicated that rises in the plasma concentrations of CK are primarily the result of inflammation caused by muscle damage following unaccustomed exercise, which can induce delayed onset muscle soreness after 6–8 h [39,40]. Burns et al. claimed that the inflammation caused by muscle damage is the main cause of postprandial plasma lipid level increases [41]; however, Pafili and colleagues argued that the high plasma concentrations of CK are caused by eccentric muscle actions and do not affect the postprandial TG concentrations [42]. This is probably because additional energy is required after exercise to repair damaged tissues, which results in higher energy expenditure [43]. However, this mechanism requires further investigation. Nevertheless, the present study indicated that the raised CK concentrations caused by post-HIIE muscle inflammation did not reduce the ability of HIIE to lower the postprandial TG concentrations.

In the present study, the postprandial TG concentrations in the MICE and CON exhibited no significant difference, confirming the findings of numerous studies. For example, Petitt et al. found the no significant differences were detected in postprandial TG concentrations between MICE at an energy expenditure of 382.2 kcal and a control trial [38]. Moreover, Tan and colleagues required participants to engage in bicycle riding at 70% VO2max for 20 min with an energy expenditure of 173 kcal [12], and Gabriel and colleagues required participants to walk for 30 min at an energy expenditure of 240.9 kcal. In both of these studies, MICE did not reduce the postprandial TG levels the following day. In the present study, the MICE lasted for approximately 36 min, with an energy expenditure of 384.4 ± 69.9 kcal. Similarly, the postprandial TG concentrations the following day were unaffected. The decrease in postprandial TG concentrations may have been due to post-exercise energy replenishment [10], the CHO contents in high fat meals [11] or the mode and intensity of exercise. However, the data in the present study were insufficient to clearly determine the reasons that MICE does not effectively influence the postprandial TG concentrations.

## 5. Limitations

The present study had two limitations: First, because a muscle biopsy was not implemented, this study was unable to reveal the exact muscle glycogen and IMTG levels before and after exercise. However, studies have clearly indicated that HIIE is more capable of lowering muscle glycogen than is MICE. Furthermore, in the present study, the participants reached the average HR of 140.1 ± 3.7 beats, meeting the standards for high-intensity exercise. Second, because the post-exercise EPOC was not measured, the energy expenditure 24 h prior to the high-fat meal was not accurately determined. Future studies are advised to further investigate the energy expenditure 24 h before the high-fat meal and its effect on the postprandial TG concentrations.

## 6. Conclusions

The present study revealed that among exercise interventions with different intensities and the same energy expenditure, HIIE is more capable of reducing the postprandial TG concentrations. This may be attributed to the effectiveness of HIIE at raising the postprandial fat oxidation rate. Additionally, the present study also indicated that HIIE-induced muscle damage could drastically increase the concentration of CK, an inflammatory indicator. However, this phenomenon does not attenuate the ability of HIIE to reduce the postprandial plasma lipid level. The findings of the present study are expected to provide valuable reference for the design of exercise prescription for people in need of dietary and exercise intervention to improve body composition.

## Figures and Tables

**Table 1:** Exercise indices.

	HIIE	MICE
Body fat (%)	21.8 ± 6.2	
VO2max (mL/min/kg)	42.2 ± 7.86	
Mean heart rate (bpm)	140.1 ± 3.7	127.8 ± 1.9
Max heart rate (bpm)	171.0 ± 9.1	138.7 ± 2.0
Exercise time (min)	20	36.6 ± 4.8
Slope (%)	NA	4.9 ± 2.6
Speed (km/h)	NA	5.6 ± 0.2
EE (kcal)	384.4 ± 69.9	376.7 ± 76.9

Values are the mean SD (standard deviation), *n* = 9. HIIE, high intensity intermittent exercise; MICE, moderate intensity and continuous exercise; EE, energy expenditure.

**Table 2:** Plasma concentrations in the fasted state at the baseline on Day 2 of each trials.

	HIIE	MICE	CON	*p*
TG (mmol/L)	0.74 ± 0.27	0.99 ± 0.46	0.95 ± 0.31	0.174
NEFA (mmol/L)	0.54 ± 0.19	0.48 ± 0.22	0.62 ± 0.35	0.499
Glycerol (µmol/L)	37.11 ± 17.53	37.44 ± 19.84	42.56 ± 24.90	0.721
CK (mmol/L)	242.11 ± 108.62 ^1^	164.78 ± 61.03	127.78 ± 33.55	0.04
Fat oxidation (g/min)	0.07 ± 0.04	0.06 ± 0.05	0.07 ± 0.04	0.403

^1^ Significant difference between HIIE and CON (*p* < 0.05). Values are the mean SD, *n* = 9. HIIE, high intensity intermittent exercise; MICE, moderate intensity and continuous exercise; CON, control; TG, triglyceride; NEFA, non-esterified fatty acids; CK, creatine kinase.
